# Female heterozygotes for the hypomorphic R40H mutation can have ornithine transcarbamylase deficiency and present in early adolescence: a case report and review of the literature

**DOI:** 10.1186/1752-1947-4-361

**Published:** 2010-11-12

**Authors:** Jason R Pinner, Mary-Louise Freckmann, Edwin P Kirk, Makoto Yoshino

**Affiliations:** 1 Department of Molecular and Clinical Genetics, Royal Prince Alfred Hospital, Sydney Australia; 2 Department of Medical Genetics, Sydney Children's Hospital, Sydney, Australia; 3 Department of Paediatrics and Child Health, Kurume University School of Medicine, Kurume, Japan

## Abstract

**Introduction:**

Ornithine transcarbamylase deficiency is the most common hereditary urea cycle defect. It is inherited in an X-linked manner and classically presents in neonates with encephalopathy and hyperammonemia in males. Females and males with hypomorphic mutations present later, sometimes in adulthood, with episodes that are frequently fatal.

**Case presentation:**

A 13-year-old Caucasian girl presented with progressive encephalopathy, hyperammonemic coma and lactic acidosis. She had a history of intermittent regular episodes of nausea and vomiting from seven years of age, previously diagnosed as abdominal migraines. At presentation she was hyperammonemic (ammonia 477 μmol/L) with no other biochemical indicators of hepatic dysfunction or damage and had grossly elevated urinary orotate (orotate/creatinine ratio 1.866 μmol/mmol creatinine, reference range <500 μmol/mmol creatinine) highly suggestive of ornithine transcarbamylase deficiency. She was treated with intravenous sodium benzoate and arginine and made a rapid full recovery. She was discharged on a protein-restricted diet. She has not required ongoing treatment with arginine, and baseline ammonia and serum amino acid concentrations are within normal ranges. She has had one further episode of hyperammonemia associated with intercurrent infection after one year of follow up. An R40H (c.119G>A) mutation was identified in the ornithine transcarbamylase gene (*OTC*) in our patient confirming the first symptomatic female shown heterozygous for the R40H mutation. A review of the literature and correspondence with authors of patients with the R40H mutation identified one other symptomatic female patient who died of hyperammonemic coma in her late teens.

**Conclusions:**

This report expands the clinical spectrum of presentation of ornithine transcarbamylase deficiency to female heterozygotes for the hypomorphic R40H *OTC *mutation. Although this mutation is usually associated with a mild phenotype, females with this mutation can present with acute decompensation, which can be fatal. Ornithine transcarbamylase deficiency should be considered in the differential diagnosis of unexplained acute confusion, even without a suggestive family history.

## Introduction

Ornithine transcarbamylase deficiency (OTCD, online mendelian inheritance in man: OMIM #311250) is the most common hereditary urea cycle defect and is inherited in an X-linked manner [1,2]. It classically presents with encephalopathy and hyperammonemia, manifesting as tachypnea, lethargy, nausea, vomiting, behavioral changes, confusion, encephalopathy, seizures, coma, and death. There is a wide phenotypic spectrum from neonatal hyperammonemic coma to asymptomatic adults [3-5]. Male hemizygotes with null alleles present with fatal hyperammonemic neonatal encephalopathy. Treatment with survival in this group results in severe neurological impairment in almost all cases [6]. Males with hypomorphic mutations and female heterozygotes usually present later, some not until late adulthood [5,7-9]. Approximately 15 to 20% of female heterozygotes have encephalopathic episodes, a significant proportion of which are fatal [10]. Later onset patients may, in retrospect, have a history of self-restricted protein intake or episodes of nausea and vomiting due to transient hyperammonemia. Typical triggers are excessive protein load, including total parenteral nutrition in previously undiagnosed individuals [11], catabolic stress, the postpartum period [12], and certain drugs, notably sodium valproate [13]. The diagnosis can be made on elevated serum ammonia with high urinary orotate. The aim of treatment of episodes of decompensation is to reduce ammonia levels with sodium benzoate or phenylbutyrate that increase alternate ammonia excretion, arginine replacement and protein restriction. Hemodialysis or hemofiltration is indicated in severe cases. Future episodes are reduced with dietary protein restriction, oral benzoate or phenylbutyrate and arginine supplementation.

## Case presentation

Our patient was a previously well 13-year-old Caucasian girl, referred to our metabolic service from intensive care because of unexplained encephalopathy and lactic acidosis. She presented to the emergency department with a two-day history of vomiting and deteriorating mentation, progressing to confusion. Initial physical examination was unremarkable except for decreased conscious level. There was no hepatomegaly or signs of chronic liver disease. Initial venous blood gas analysis demonstrated a respiratory alkalosis (pH 7.488, pCO_2 _29.9 mmHg, bicarbonate 22.5 mmol/L, standard base excess -0.5 mmol/L), mild lactic acidemia (lactate 2.6 mmol/L) and normoglycemia (glucose 5.7 mmol/L). Her alanine aminotransferase (ALT) was mildly elevated at 53 U/L (reference range <45 U/L) but other liver function tests, international normalized ration (INR) and activated partial thromboplastin time (APTT), electrolytes, urea, creatinine, amylase, lipase, full blood count, erythrocyte sedimentation rate (ESR), and C-reactive protein, were all within the normal ranges. There was no history of drug or toxin ingestion and a urinary drug screen did not detect any amphetamines, cocaine, cannabis or opiates. A lumbar puncture and cerebral computed tomography (CT) scan were normal. She was admitted to the intensive care unit for observation because of her decreased level of consciousness, commenced on intravenous fluids (0.45% saline and 5% dextrose) and broad-spectrum antimicrobials to cover sepsis and central nervous system infection after appropriate cultures had been taken. She became increasingly agitated and confused, developed opisthotonos and a significant lactic acidosis without hypoglycemia (pH 7.387, pCO_2 _32.1 mmHg, bicarbonate 18.9 mmol/L, standard base excess -5.2 mmol/L, glucose 8.2, lactate 9.5 mmol/L). Serum ammonia was 477 μmol/L, suggesting a urea cycle defect. She was treated with intravenous sodium benzoate (loading dose of 250 mg/kg followed by an infusion at 250 mg/kg/day), arginine (200 mg/kg/day), 10% dextrose and lipid infusions. Biochemical markers improved rapidly, with the ammonia falling to 208 μmol/L within three hours. The urine metabolic screen showed a gross elevation in orotate (orotate/creatinine ratio 1.866 μmol/mmol creatinine, reference range <500 μmol/mmol creatinine), and plasma quantitative amino acid analysis showed a typical picture associated with OTCD. Clinically she became more alert and recovered with normal mentation over four to five days. A large protein load consumption associated with festivities was subsequently identified as the trigger for her decompensation. She was put on a protein-restricted diet at maximum protein intake of 2 g/kg/day. A detailed dietary history revealed that she had been subtly self-restricting her protein intake to approximately this level beforehand.

Further history elucidated frequent episodes of nausea and vomiting from about seven years of age, two to three times per week, particularly after restaurant meals or after exercise. There were also shorter episodes of nausea relieved after vomiting. A gastroenterologist had made a diagnosis of abdominal migraines. Psychomotor development was normal: she was in the appropriate academic year at school, was well coordinated and a good athlete. She is the youngest of four children born to non-consanguineous Caucasian parents. Her mother experienced migraines. A great uncle through the maternal line died as a baby of unknown cause. No relatives were known to have had similar episodes of nausea, vomiting or confusion. Cascade testing of other family members after appropriate genetic counseling is proceeding.

Dietary protein restriction has resulted in no recurrence of her episodes of nausea and vomiting. Her serum amino acids and ammonia levels have been within normal ranges on dietary restriction alone and she has not required oral treatment with phenylbutyrate or arginine.

The family was given a written emergency plan. She has had one episode of mild hyperammonemia (maximum ammonia 105 μmol/L) associated with vomiting due to a gastrointestinal illness, from which she made a full recovery. She has also had an episode of viral myositis since her initial admission, without developing neurological symptoms or signs. She shows no demonstrable adverse effects on her growth or development.

## Discussion

A review of the literature identified another family with symptomatic females presumed carriers for the R40H *OTC *mutation. The index case was a 17-year-old Japanese boy who presented with a history of several days of nausea and vomiting, became confused, developed depressed level of consciousness, then seizures and died on the second day of hospital admission [7]. He was posthumously shown to have the R40H mutation [14]. The family history revealed that his younger sister had experienced two episodes of nausea and vomiting aged 13 each lasting one week [7]. The mother was asymptomatic but had mild protein load induced orotic aciduria. Clinical follow-up of the younger sister was complicated by suboptimal compliance. Unfortunately, she died aged 18 after hyperammonemic coma, having presented after festive protein ingestion. It has not been possible to obtain molecular confirmation that she and her mother are manifesting carriers of the R40H *OTC *mutation. However, her clinical course, her mother's biochemical investigations and family history strongly suggest this conclusion.

Manifestations of OTCD are highly variable and depend upon the severity of the mutation and sex of the individual. The classic presentation is males with hemizygous null mutations with no residual enzyme activity who present in the neonatal period with progressive metabolic encephalopathy due to hyperammonemia with death in the first week of life. Milder mutations with varying degrees of residual enzyme activity produce later onset from the first year of life to adulthood. Some adults remain apparently asymptomatic, with protein aversion their only symptom. Female heterozygotes have an even wider spectrum of disease due to variable X-inactivation in the liver. Specific triggers for encephalopathic episodes are high protein loads, pregnancy and certain drugs, including valproate. It is important to recognize OTCD in later onset cases as the first presentation is frequently fatal [10].

The risk of female heterozygotes developing symptoms is difficult to quantify, posing practical problems for management. Historical data are limited since diagnosis is generally through affected males, suggesting that female heterozygotes infrequently manifest symptoms. X-inactivation studies on blood may not truly reflect hepatic skewing and are therefore not reliable in predicting risk of hyperammonemic episodes in female heterozygotes [15]. X-inactivation studies on liver biopsies might give more information but carry the risk of procedure related morbidity. Our approach when counseling family members has been to offer at risk males and females genetic testing, regardless of age, and to take a history specifically for exposure to significant known triggers.

## Conclusions

Females heterozygous for the R40H *OTC *mutation should be considered at risk for hyperammonemic episodes, and should have appropriate counseling, biochemical surveillance and treatment, institution of an emergency treatment plan to prepare for possible hyperammonemic episodes and monitoring during the postpartum period.

More generally, for patients presenting with unexplained confusion or decreased level of consciousness, urea cycle defects and in particular OTC should be considered in the differential diagnosis and an urgent ammonia level determined.

## List of abbreviations

ALT: alanine amino transferase; APTT: activated partial thromboplastin time; CT: computed tomography; ESR: erythrocyte sedimentation rate); INR: international normalized ratio; OMIM: online mendelian inheritance in man; OTC: ornithine transcarbamylase.

## Competing interests

The authors declare that they have no competing interests.

## Consent

Written informed consent was obtained from the patient and their parent for publication of this case report and any accompanying images. Copies of the written consents are available for review by the journal's Editor-in-Chief. Written informed consent was previously obtained from the patients and/or their parents for the original publication of the case discussed in the literature review.

## Authors' contributions

JP wrote the manuscript with comments and revision by EK, MLF and MY. MLF was the managing clinician at presentation, and EK the physician continuing care. All the authors read and approved the final manuscript.
